# Bone Marrow Aspirate Concentrate Combined With Demineralized Bone Matrix With Open Curettage vs Mini-Open Trocar-Assisted Injection for Unicameral Bone Cysts: A Retrospective Comparative Study

**DOI:** 10.1016/j.jposna.2026.100449

**Published:** 2026-08-26

**Authors:** Roy Gigi, Amir Sternheim, Omri Merose, Adrian Tudor, Ortal Segal, Baruch Danino, Yair Gortzak

**Affiliations:** 1 Pediatric Orthopaedic Department, Dana-Dwek Children's Hospital, Tel Aviv Sourasky Medical Center, Tel Aviv, Israel; 2 Orthopaedic Oncology Department, Tel Aviv Sourasky Medical Center, Tel Aviv, Israel; 3 Department of Orthopedic Surgery, Gray School of Medicine, Gray Faculty of Medical and Health Sciences, Tel Aviv, Israel

**Keywords:** Unicameral bone cyst, Bone marrow aspirate, Concentrate, Demineralized bone matrix, Open curettage, Pediatric orthopaedic oncology

## Abstract

**Background:**

Unicameral bone cysts (UBCs) are common benign pediatric bone lesions prone to pathologic fracture and recurrence. Bone marrow aspirate concentrate combined with demineralized bone matrix (BMAC/DBM) is a biologically active treatment strategy. However, no study has directly compared open curettage combined with BMAC/DBM injection against mini-open trocar-assisted injection (MOTAI) of BMAC/DBM in a long-term cohort.

**Methods:**

A retrospective comparative study was conducted at a single tertiary referral center. Thirty-five patients treated for UBCs between 2006 and 2019 were included: 20 underwent open curettage with BMAC/DBM injection (curettage group) and 15 received MOTAI (MOTAI group). The primary outcome was the need for an additional surgical intervention. Secondary outcomes included final Neer radiographic classification and a composite functional score at a mean follow-up of 7.8 years.

**Results:**

The reoperation rate was 15.0% (3/20) in the curettage group vs 66.7% (10/15) in the MOTAI group (Fisher’s exact *P* = .028 after adjustment for age and cyst activity status). On multivariable logistic regression, the MOTAI group had significantly higher odds of undergoing an additional surgical intervention than the curettage group (odds ratio: 9.92, 95% confidence interval: 1.27-77.35 *P* = .029). Cyst activity status did not independently predict the need for an additional intervention (*P* = .394). A good radiographic result (Neer grades 1-2) was achieved in 70.0% of the curettage group and 73.3% of the MOTAI group (Fisher’s exact *P* = 1.00). Hardware use did not significantly influence reoperation risk (*P* = .835). Composite functional scores were excellent in both groups, with no significant difference between them (Benjamini-Hochberg–adjusted *P* > .80).

**Conclusions:**

Open curettage with BMAC/DBM significantly reduces the need for additional surgical interventions compared with MOTAI, without compromising the final radiographic or functional outcomes at long-term follow-up. Curettage may be the principal surgical determinant of treatment efficacy in this setting.

**Key Concepts:**

(1) Open curettage with bone marrow aspirate concentrate combined with demineralized bone matrix lowers reoperation risk vs injection alone in unicameral bone cysts (UBCs).
(2) Long-term radiographic and functional outcomes were similar between approaches.
(3) Intramedullary hardware alone did not reduce reoperation risk in pediatric UBCs.
(4) Active cyst status did not independently predict reoperation after adjustment.
(5) Mechanical cyst-wall disruption may be the key surgical driver of cyst healing.

**Level of Evidence:**

III, Retrospective Comparative Study

## Introduction

Unicameral bone cysts (UBCs), also known as simple bone cysts, are benign, fluid-containing, single-chamber osseous lesions that predominantly affect the pediatric skeleton, accounting for approximately 3% of all primary bone tumors [1,2]. First described by Virchow in 1876, they most commonly arise in the metaphysis of long bones, with a predilection for the proximal humerus (55%-65%) and proximal femur (25%-30%) [3,4]. The mean age at diagnosis is approximately 9 years, with a male predominance of roughly 2:1 [1,5].

UBCs do not possess a true secretory cyst wall similar to that of a ganglion cyst. Instead, they are thought to result from a localized disturbance in bone remodeling, with the thin fibrous lining representing a secondary reactive membrane rather than a causative structure [6]. The clinical behavior of UBCs is closely related to patient age and lesion activity. Active cysts, defined by their location within 1 cm of the physis, have a recurrence rate nearly 4 times higher than that of latent lesions and are associated with progressive cortical thinning and an increased risk of pathologic fracture [5,7]. Approximately 80% of patients present following a pathologic fracture, whereas others are diagnosed incidentally or during evaluation for mild pain [2,8]. With skeletal maturation, lesions typically migrate toward the diaphysis and become biologically inactive; however, a substantial proportion fails to resolve spontaneously and may ultimately require surgical intervention.

Owing to their benign nature and the possibility of spontaneous resolution, establishing standardized indications and the optimal extent of surgical intervention remains challenging [9]. Surgery is generally reserved for symptomatic lesions, high fracture risk (large or active lesions with cortical thinning), fractures in weight-bearing bones, radiographically progressive cysts, or cases requiring histologic confirmation [10]. The principal goals of treatment are fracture reduction, cyst resolution, and recurrence prevention [9].

Multiple treatment strategies have been described, including corticosteroid injection, autologous bone marrow (BM) injection, demineralized bone matrix (DBM) grafting, calcium-based substitutes, open curettage with bone grafting, and intramedullary stabilization [[11], [12], [13], [14], [15], [16], [17]]. Corticosteroid injection yields healing rates of only 41%-63% after a single injection [11,12]. Comparative series have evaluated steroid injection against autologous BM grafting [14] and against multiple surgical alternatives [15], with variable success rates. BM injection exploits the osteogenic potential of marrow-derived mesenchymal stem cells (MSCs), and bone marrow aspirate concentrate (BMAC) combined with DBM (BMAC/DBM) has produced encouraging results [6,[18], [19], [20], [21]]. Nonetheless, recurrence and reoperation rates remain substantial across all modalities. A key unresolved question is whether the mechanical component of surgery, specifically the extent of curettage and disruption of the cyst wall, independently influences outcomes.

At our institution, 2 surgical teams managed UBCs during the study period using distinct protocols. Patients were treated with either open curettage or mini-open trocar-assisted injection (MOTAI) of BMAC/DBM: one team performed MOTAI alone; the other combined open curettage with BMAC/DBM. This institutional variation, with an identical biological substrate in both groups, provided an opportunity to isolate the contribution of curettage.

We therefore conducted a retrospective comparative study with the primary aim of determining whether open curettage with BMAC/DBM reduces fracture risk and reoperation rate compared with MOTAI. Secondary aims were to compare final radiographic outcome (Neer classification) and functional results and to evaluate independent predictors of outcome.

## Materials and methods

### Study design and patient selection

A single-center retrospective comparative study was performed using a prospectively maintained institutional database. All patients surgically treated for UBCs between 2006 and 2019 who received BMAC/DBM as part of their treatment protocol were identified. Of 46 patients initially screened, 35 fulfilled inclusion criteria.

Patients were excluded if they (1) underwent nonoperative management, (2) had fewer than 12 months of postoperative follow-up, (3) had incomplete operative records, (4) presented for treatment of a recurrent UBC initially treated at an outside institution, or (5) did not receive BMAC/DBM at the index procedure.

The 35 included patients were allocated to two groups according to the treating surgical team and its established protocol: the curettage group (open curettage followed by BMAC/DBM injection, *n* = 20) and the MOTAI group (MOTAI alone, *n* = 15).

### Surgical techniques

#### Open curettage with BMAC/DBM (curettage group)

Surgery was performed under general anesthesia and fluoroscopic guidance. A cortical bone window was created using a burr drill, and the cyst lining was meticulously removed using sharp straight and angled curettes. Specimens were submitted for histopathologic examination. Additional mechanical debridement was performed using a rose-head burr in most cases. Deliberate communication between the cyst cavity and the intramedullary canal was established in all cases. In patients with unstable pathologic fractures or those requiring additional stability, elastic stable intramedullary nailing or plate fixation was added. Sixty milliliter of bone marrow aspirate (BMA) was harvested from the iliac crest, centrifuged to yield BMAC, and injected together with DBM into the prepared cavity [22].

#### MOTAI of BMAC/DBM alone (MOTAI group)

Procedures were performed under general anesthesia and fluoroscopic guidance. A 1-cm skin incision was made at the level of the cyst, and a core trocar was advanced through the cortex into the cyst. The inner trocar was withdrawn to drain cyst fluid. A mixture of BMAC (60 mL) and DBM was then injected into the cyst cavity under fluoroscopy. Where additional stabilization was deemed necessary, intramedullary hardware was placed.

Postoperative management was standardized by anatomical site. Upper-limb patients were immobilized in a sling for 4-6 weeks, followed by progressive rehabilitation. Lower-limb patients maintained toe-touch weight-bearing for 4 weeks, advancing as permitted by clinical and radiographic healing.

### Outcome measures

The primary outcome was the need for more than 1 surgical procedure (reoperation rate). Secondary outcomes were (1) final radiographic result by the modified Neer classification [23], dichotomized as good (grades 1-2: complete or partial healing) vs suboptimal (grades 3-4: persistent cyst or recurrence) and (2) a composite functional score (maximum 9 points) comprising function, pain, and range of motion (ROM) domains (each scored 1-3), assessed at the final follow-up. Cyst activity was defined as lesion location within 1 cm of the physis [5,7].

Radiographic recurrence was determined at each follow-up visit by comparing the current radiograph with the radiograph obtained at the prior visit, based on interval change in the modified Neer classification or the treating surgeon’s overall assessment of the radiographs. Reoperation was a separate, subsequent clinical decision made at a later stage, based on a combination of the treating surgeon’s clinical and radiographic assessment at follow-up together with discussion at surgical team consultation. The specific procedure selected for a second intervention, including whether it replicated the index technique or crossed over between MOTAI and open curettage, was determined at the discretion of the treating surgical team.

#### Radiographic assessment

Neer grading was performed at each follow-up visit in conjunction with clinical evaluation, and this combined assessment informed the treating team’s discussions with patients and families regarding continued observation vs repeat intervention. The Neer grade used to report study outcomes was obtained at the final follow-up visit. All final plain radiographs were independently reviewed and graded according to the modified Neer classification by the treating senior surgeon. Assessors were either a board-certified pediatric orthopaedic surgeon or a pediatric orthopaedic oncology surgeon, each with at least 10 years of postfellowship experience and subspecialty fellowship training. Grading was blinded to treatment group assignment. In cases of discordance between the surgeon’s intraoperative impression and the radiographic grade, the radiographic classification was used as the definitive outcome.

#### Functional outcome assessment

A composite clinical score (maximum 9 points) was constructed from 3 equally weighted domains: limb function, pain at rest and during activity, and ROM of the affected joint. Each domain was scored on a 3-point ordinal scale, as detailed in Table 1. Scores were assigned at the final follow-up by the treating surgeon based on clinical examination and structured patient interview. A total score of 7-9 was classified as excellent, 4-6 as good, and ≤3 as poor, consistent with prior UBC outcome studies [20,21]. Although not formally validated, the score was prospectively recorded by the treating surgeon at each visit based on clinical examination and structured interview, with the final follow-up score used for analysis.

**Table 1:** Composite functional scoring criteria.

Domain	Score: 1—poor	Score: 2—moderate	Score: 3—good
Limb function	Unable to perform activities of daily living with significant functional limitation	Able to perform ADLs with some restriction unable to return to full sport or prior activity level	Full return to preinjury activity level including sport no functional restriction
Pain	Persistent pain at rest or with minimal activity requires regular analgesia	Pain only with strenuous activity or loading no analgesia at rest	Pain-free at rest and during all activities no analgesia required
Range of motion	ROM < 50% of contralateral side	ROM: 50-80% of contralateral side	ROM > 80% of contralateral side

*ADLs, activities of daily living; ROM, range of motion*.

*Scoring: Each domain is scored 1-3, and the 3 domain scores are summed (range: 3-9). Total score interpretation: 7-9 = excellent; 4-6 = good; ≤3 = poor.*

### Statistical analysis

Continuous variables, including age at surgery and the composite functional score, were compared using the Mann-Whitney *U* test as these measures did not meet assumptions of normality. Categorical variables, including surgical group, hardware use, active cyst status, anatomical site, and reoperation, were analyzed using the Pearson’s chi-squared or Fisher’s exact test when expected cell counts were small. Both raw *P* values and Benjamini-Hochberg false discovery rate (BH-FDR)–adjusted *P* values are reported. Statistical inferences are based on adjusted values. Associations significant before but not after FDR correction were interpreted conservatively as nonsignificant.

Two multivariable logistic regression models were constructed. Model A evaluated independent predictors of reoperation, with surgical approach (curettage vs MOTAI) as the primary covariate, adjusted for active cyst status. Model B evaluated predictors of achieving a good Neer classification, adjusted for age, cyst activity, hardware use, and anatomical site. Results are presented as odds ratios (ORs) with 95% confidence intervals (CIs). All tests were 2-sided (significance level *P* < .05). Analyses were performed using IBM SPSS Statistics 29.0.1.0 (IBM Corp., Armonk, NY, USA) and R 4.4.0 (R Foundation for Statistical Computing, Vienna, Austria).

## Results

### Patient demographics and baseline characteristics

Thirty-five patients (25 males and 10 females; M:F = 2.5:1) were included. Mean age at diagnosis was 7.9 ± 3.5 years (range: 2.5-14.9), and mean age at surgery was 9.1 ± 3.9 years (range: 2.9-18.2). Mean follow-up was 7.8 years (median: 7.9; range: 1.6-12.4). Baseline characteristics are summarized in Table 2. The humerus was the most common site (22/35, 62.9%), followed by the femur (10/35, 28.6%). Fracture at diagnosis was present in 20 of 35 patients (57.1%). Patients with an active cyst were significantly younger than those with a latent cyst (Mann-Whitney U, *P* = .017).

**Table 2:** Baseline characteristics of the curettage and MOTAI groups.

Characteristic	Curettage group (*n* = 20)	MOTAI group (*n* = 15)	*P* value	Significance
***Demographics***				
Sex (male:female)	13:7	12:3	.458	*ns*
Age at diagnosis, mean ± SD (yr)	8.3 ± 2.6	7.3 ± 4.4	.309	*ns*
Age at surgery, mean ± SD (yr)	9.8 ± 3.2	8.1 ± 4.5	.271	*ns*
Follow-up, mean ± SD (yr)	8.1 ± 2.9	7.4 ± 2.6	.172	*ns*
***Anatomic site*, *n* (%)**				
Humerus	14 (70.0%)	8 (53.3%)	.267	*ns*
Femur	4 (20.0%)	6 (40.0%)	–	–
Other	2 (10.0%)	1 (6.7%)	–	–
***Clinical features*, *n* (%)**				
**Active cyst (within 1 cm of physis)**	**5 (25.0%)**	**11 (73.3%)**	**.005**	**∗∗*P* < .01**
Fracture at diagnosis	14 (70.0%)	6 (40.0%)	.087	*ns*
**Hardware used at index surgery**	**2 (10.0%)**	**7 (46.7%)**	**.022**	**∗*P* < .05**

*MOTAI, mini-open trocar-assisted injection; SD, standard deviation*.

***Statistical tests***: *Continuous variables were compared using the Mann-Whitney U test. Categorical variables were compared using the Pearson’s chi-squared or Fisher’s exact test (where expected counts were <5).****Multiple-comparison correction***: *Benjamini-Hochberg (BH) false-discovery-rate adjustment applied.****Significance***: *ns = not significant ∗BH-adjusted P < .05 ∗∗ BH-adjusted P < .01. Significant rows are highlighted in bold. Two clinically meaningful baseline imbalances were observed: more patients in the MOTAI group had active cysts and received hardware at the index surgery. These covariates were carried forward into the multivariable models* (Table 4).

The two groups were well matched for age, sex, anatomical site, and fracture prevalence. Two important pretreatment differences existed. First, active cysts were significantly more prevalent in the MOTAI group (11/15, 73.3%) than in the curettage group (5/20, 25.0% BH-adjusted *P* = .005). Second, hardware use was significantly higher in the MOTAI group (7/15, 46.7%) than in the curettage group (2/20, 10.0% BH-adjusted *P* = .022). These differences were addressed in the multivariable analysis as primary confounders.

### Primary outcome: reoperation rate

Across the entire cohort, the cumulative recurrence rate was 58.3% (95% CI: 41.4%-74.4%) and the rate of requiring 2 or more surgical procedures was 42.3% (95% CI: 26.4%-59.2%) (Fig. 1).

**Figure 1:**
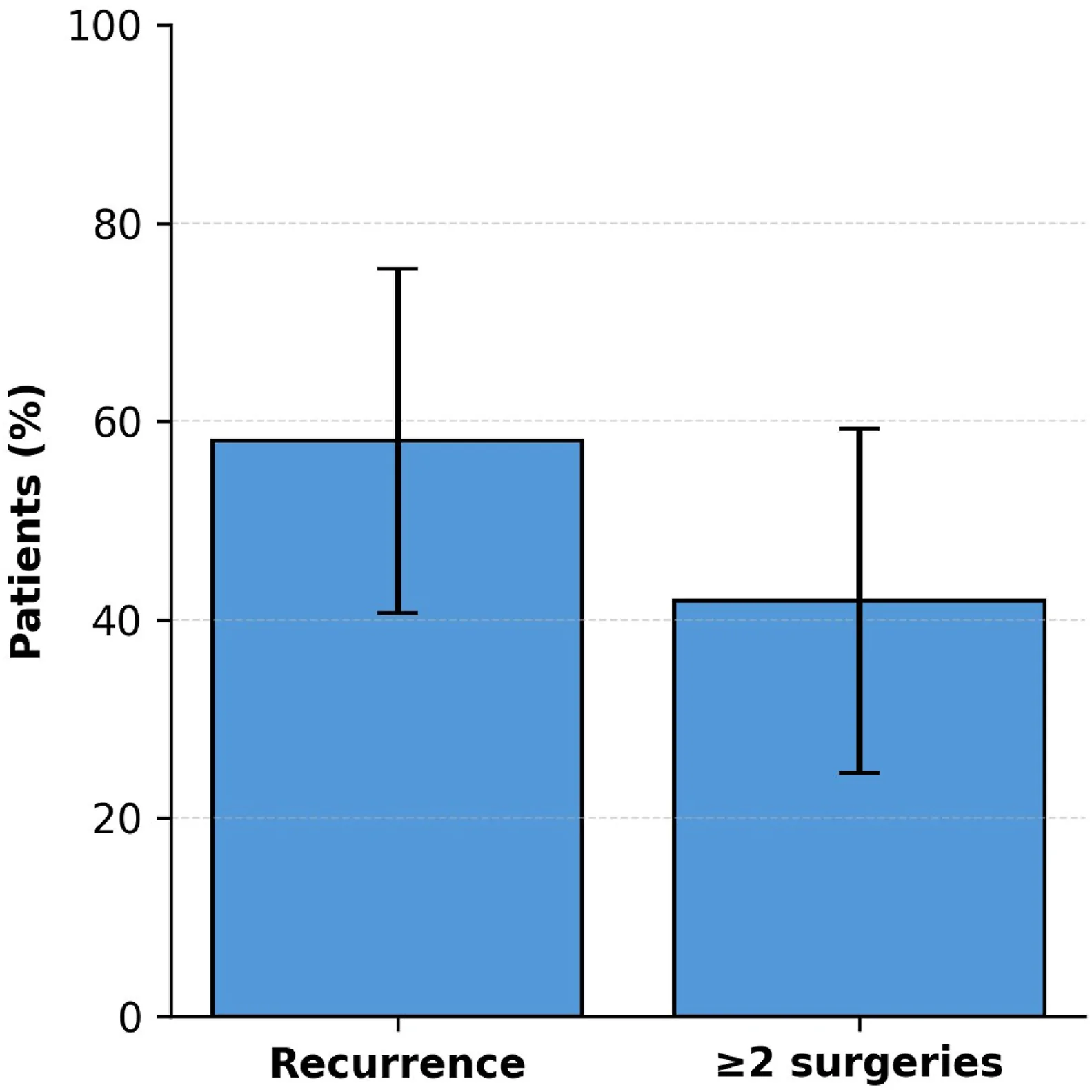
Overall cohort outcomes: cumulative recurrence rate (58.3%) and rate of requiring ≥2 surgical procedures (42.3%) with 95% confidence intervals.

The reoperation rate differed markedly between groups: 3 of 20 patients (15.0%) in the curettage group vs 10 of 15 (66.7%) in the MOTAI group (Fig. 2). The difference remained statistically significant after multivariable adjustment for age and active cyst status (Fisher’s exact *P* = .028) (Fig. 3).

**Figure 2:**
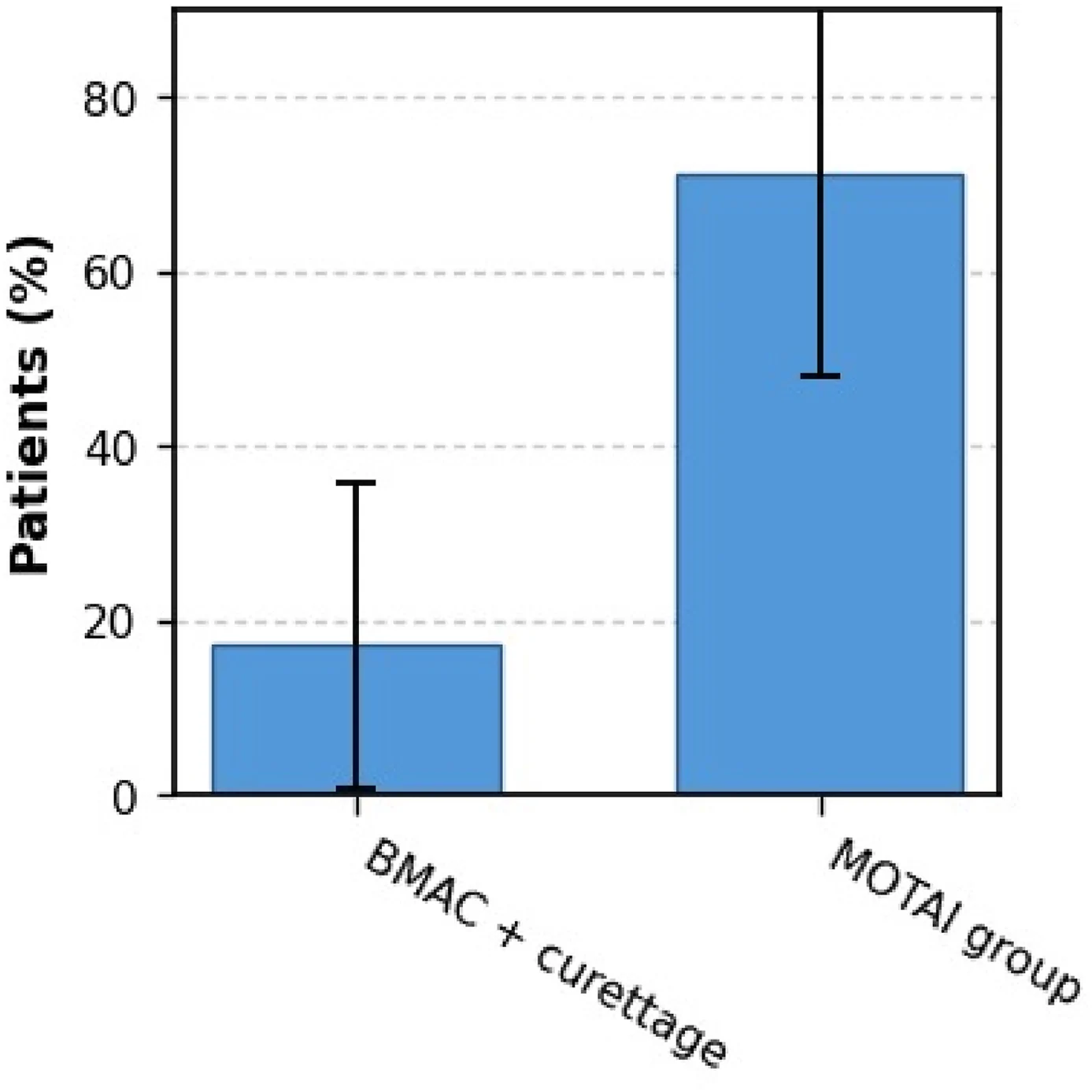
Unadjusted reoperation rates: BMAC + curettage group (15.0%) vs MOTAI group (66.7%). *BMAC, bone marrow aspirate concentrate; MOTAI, mini-open trocar-assisted injection*.

**Figure 3:**
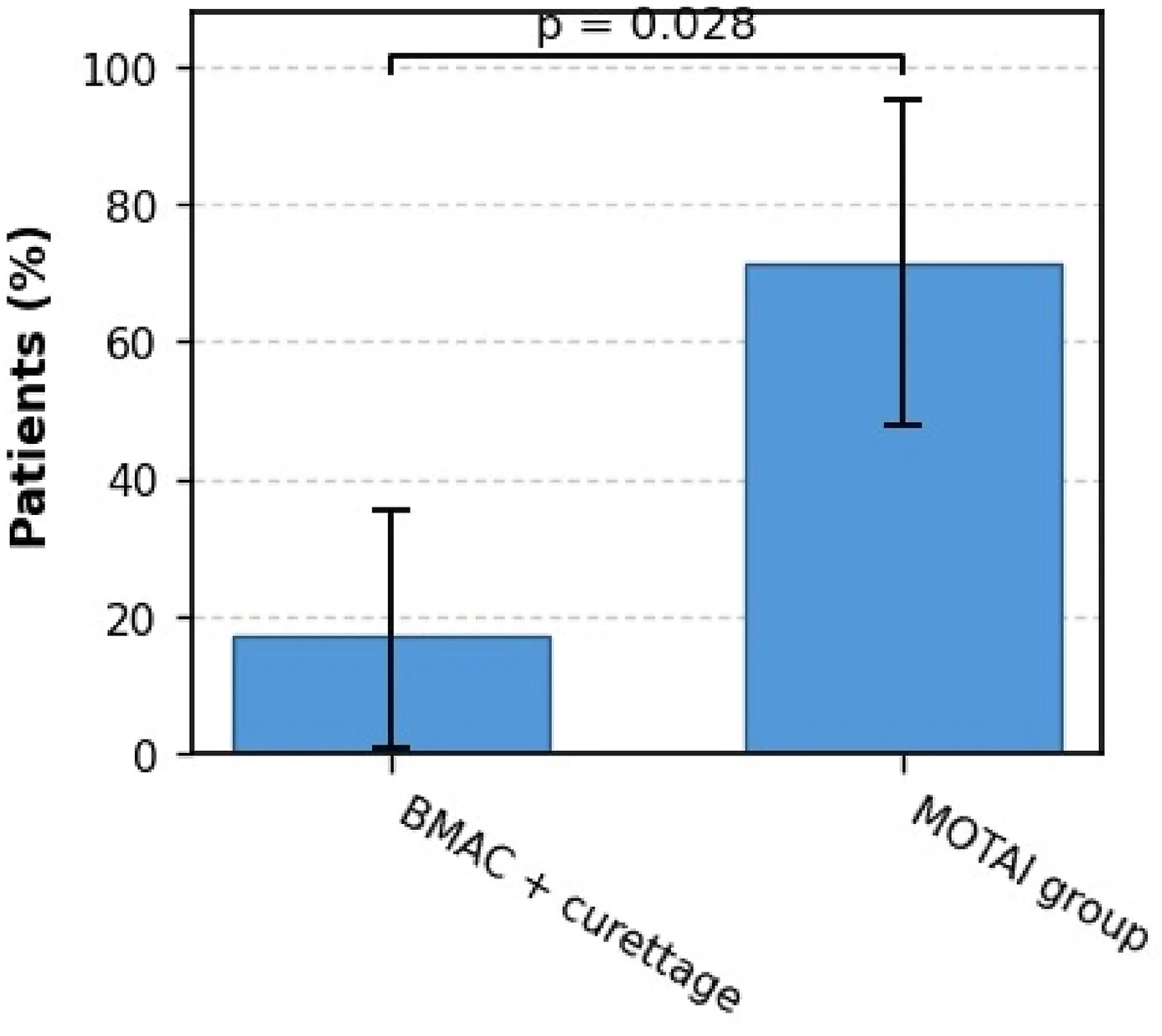
Reoperation rates after multivariable adjustment for age and active cyst status (*P* = .028): BMAC + curettage vs MOTAI. *BMAC, bone marrow aspirate concentrate; MOTAI, mini-open trocar-assisted injection*.

### Effect of hardware on reoperation

Hardware use did not significantly influence the reoperation rate (Fig. 4). After multivariable adjustment for anatomical site, age, and active cyst status, patients with hardware had a reoperation rate of approximately 45.0% compared with 41.0% for those without hardware (*P* = .835), confirming that implant placement alone does not confer an advantage in avoiding reoperation.

**Figure 4:**
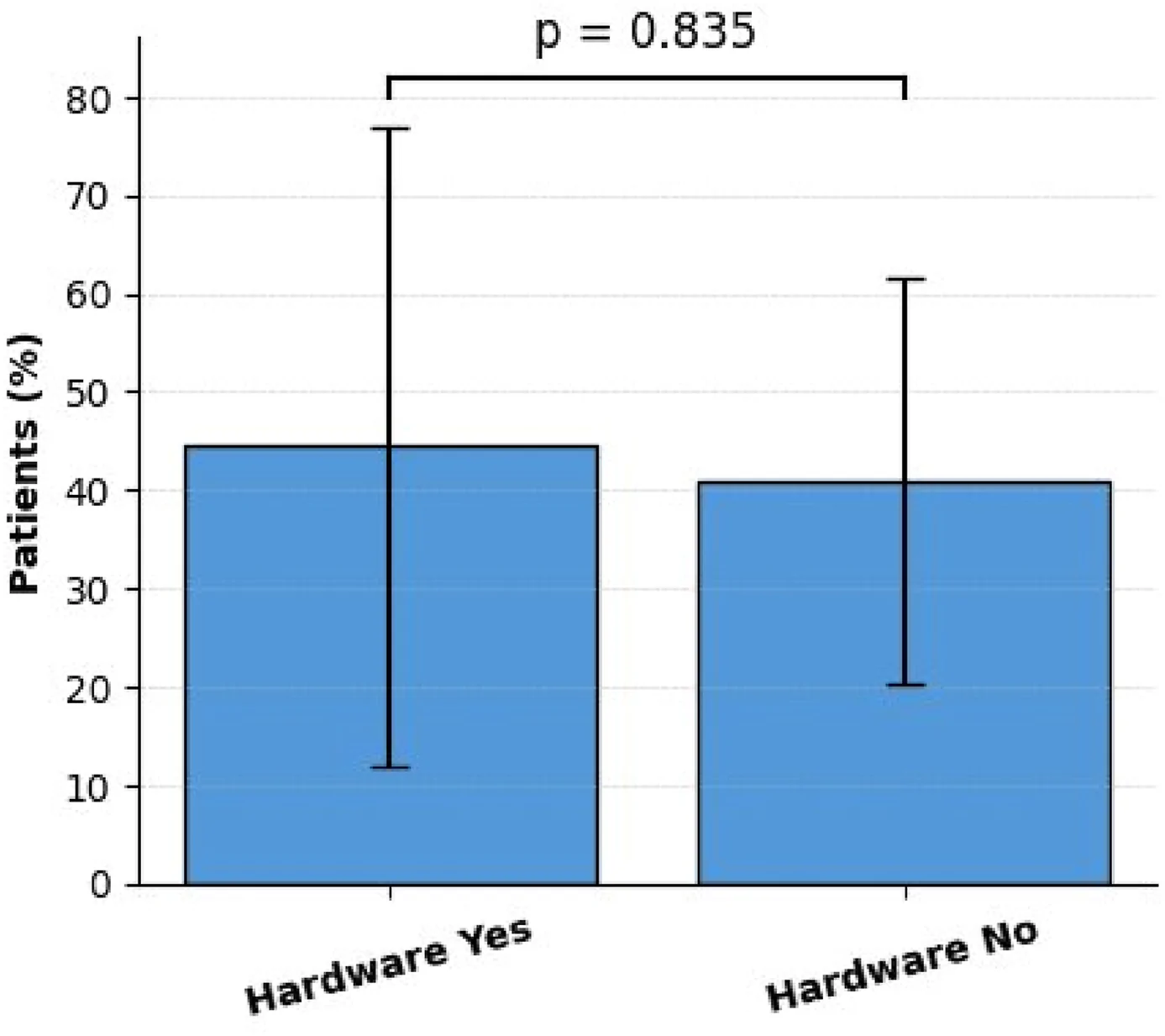
Reoperation rates stratified by hardware use after multivariable adjustment (*P* = .835): hardware vs no hardware.

### Secondary outcomes: radiographic and functional results

Final Neer classifications are summarized in Table 3. A good radiographic result (Neer grades 1 or 2) was achieved in 25 of 35 patients (71.4%). There was no significant difference between groups: 14 of 20 (70.0%) in the curettage group vs 11 of 15 (73.3%) in the MOTAI group (Fisher’s exact *P* = 1.00 Mann-Whitney *P* = .88 for Neer as a continuous variable).

**Table 3:** Primary and secondary outcomes.

Outcome	Curettage group (*n* = 20)	MOTAI group (*n* = 15)	BH-adj. *P*	Significance
***Primary outcome—****Did patients require additional surgery?*				
**Reoperation (> 1 surgical procedure)**	**3 (15.0%)**	**10 (66.7%)**	**.028**	**∗*P* < .05**
***Radiographic outcome—Neer classification.****Grade 1 = complete healing; grade 4 = recurrence.*				
Grade 1***—***complete healing	11 (55.0%)	9 (60.0%)	–	–
Grade 2***—***partial healing	3 (15.0%)	2 (13.3%)	–	–
Grade 3***—***persistent cyst	6 (30.0%)	3 (20.0%)	–	–
Grade 4***—***recurrence	0 (0.0%)	1 (6.7%)	–	–
Good result (grades 1-2 combined)	14 (70.0%)	11 (73.3%)	1.000	*ns*
***Functional outcome—****Composite score (3 domains × 3 points maximum = 9). See*Table 1*for scoring criteria.*				
Composite score, mean ± SD (max 9)	8.65 ± 0.75	8.27 ± 1.62	.836	*ns*
Limb function = 3 (max), *n* (%)	19 (95.0%)	14 (93.3%)	>.80	*ns*
Pain = 3 (max), *n* (%)	17 (85.0%)	11 (73.3%)	>.80	*ns*
Range of motion = 3 (max), *n* (%)	18 (90.0%)	14 (93.3%)	>.80	*ns*

*MOTAI, mini-open trocar-assisted injection; SD, standard deviation*.

***Statistical tests***: *Categorical variables were compared by the Pearson’s chi-squared or Fisher’s exact test. Continuous variables were compared by the Mann-Whitney U test*. ***Multiple-comparison correction***: *Benjamini-Hochberg (BH) false-discovery-rate adjustment applied across all outcomes*. ***Significance***: *ns = not significant ∗BH-adjusted P < .05 after adjustment for age and active cyst status. Significant rows are highlighted in bold. Key finding: Although radiographic healing and functional scores were similar between groups, the MOTAI group had significantly higher odds of requiring reoperation (see*Table 4*for the adjusted analysis)*.

**Table 4:** Multivariable logistic regression of factors associated with reoperation and functional outcome.

Predictor variable	Adj. OR	95% CI	*P* value	BH-adj. *P*	Interpretation
***Model A***—***outcome:reoperation (>1 surgery).****Does treatment type or cyst activity predict the need for additional surgery?*					
Active cyst status (yes vs no)	2.14	0.37-12.22	.394	.394	*No significant effect*
**MOTAI group vs curettage group**	**9.92**	**1.27-77.35**	**.029**	**.036∗**	**MOTAI group had ∼10× higher odds of reoperation**
***Model fit:****McFadden R*^*2*^ = *0.236 (acceptable fit)*					
***Model B***—***outcome:good Neer result (grades 1-2).****Which factors predict a favorable functional/radiographic outcome?*					
Age at surgery	1.01	0.78-1.30	.939	.939	*No significant effect*
Active cyst status (yes vs no)	0.89	0.14-5.70	.902	.939	*No significant effect*
Hardware use (yes vs no)	0.38	0.03-5.21	.470	.939	*No significant effect*
Femoral site (yes vs no)	10.07	0.43-233.8	.150	.600	*No significant effect (very wide CI)*
MOTAI group vs curettage group	1.47	0.32-6.84	.624	.939	*No significant effect*
***Model fit:****McFadden R*^*2*^ = *0.079 (poor fit*—*predictors explain little variance)*					

*OR, odds ratio; CI, confidence interval; BH, Benjamini-Hochberg*.

*Reference categories: Curettage group (treatment), no active cyst, no hardware, nonfemoral site. Interpretation of OR: an OR >1 indicates higher odds of the outcome than the reference OR <1 indicating lower odds. A 95% CI that crosses 1.0 indicates the effect is not statistically significant. P values were adjusted for multiple comparisons within each model using the BH false-discovery-rate procedure. ∗BH-adjusted P < .05 (statistically significant). Significant rows are highlighted in bold. The wide confidence interval for femoral site (0.43-233.8) reflects sparse data and should be interpreted with caution*.

Functional scores at the final follow-up were excellent across the cohort. The mean composite score was 8.49 ± 1.20 out of 9. No significant between-group difference was found (curettage: 8.65 ± 0.75 vs MOTAI: 8.27 ± 1.62; BH-adjusted *P* = .836). Individual domain scores for function, pain, and ROM did not differ between groups (all BH-adjusted *P* > .80).

### Logistic regression analysis

In model A (predictor of reoperation), surgical approach was the sole independent predictor of requiring more than 1 procedure. Patients in the MOTAI group had markedly higher odds of reoperation than those in the curettage group (OR: 9.92, 95% CI: 1.27-77.35 *P* = .029). Active cyst status did not independently predict the need for an additional surgical intervention after adjusting for the surgical approach (OR: 2.14, 95% CI: 0.37-12.22 *P* = .394). The Spearman correlation between the number of procedures and the final Neer score was rho = −0.18 (*P* = .31), confirming that repeat surgery did not impair long-term radiographic outcome.

In model B (predictor of good Neer result), no variable reached significance, including surgical approach (OR: 1.47, 95% CI: 0.32-6.84 *P* = .624), age, active cyst status, hardware use, or anatomical site (all BH-adjusted *P* > .18 McFadden R^2^ = 0.079). This weak overall model fit indicates that the included predictors explain little of the variance in radiographic outcome and that the nonsignificant covariates in model B should therefore be interpreted as inconclusive rather than as evidence that these factors have no true effect on radiographic outcome. Although the two groups differed substantially in how many procedures were required, they ultimately achieved equivalent radiographic healing. Regression results are summarized in Table 4.

## Discussion

This study leveraged an institutional variation in surgical practice, in which 2 teams used identical BMAC/DBM augmentation but differed in the use of open curettage, to examine the contribution of the mechanical component of treatment in pediatric UBCs. Open curettage was associated with a reduced need for repeat procedures, independent of cyst activity status and hardware use, while long-term radiographic and functional outcomes were comparable between approaches. The most clinically significant finding is the difference in reoperation rates: 15.0% in the curettage group vs 66.7% in the MOTAI group, which persisted after multivariable adjustment for cyst activity status and age (OR: 9.92, 95% CI: 1.27-77.35 *P* = .029).

The favorable mechanical and biological environment created by curettage, including systematic disruption of the cyst wall, removal of the cyst lining and necrotic tissue, and restoration of intramedullary continuity, may contribute substantially to successful healing. However, whether this benefit is attributable specifically to removal of the cyst lining or, more broadly, to mechanical decompression and stimulation of the local bone-healing response cannot be determined from the present study and remains theoretical. Nevertheless, the concept that mechanical disruption and decompression of the cyst may provide a biological advantage over percutaneous injection alone is supported by the existing literature.

Re-establishing communication between the medullary canal and the cyst cavity is thought to reduce intramedullary pressure, enhance drainage, and facilitate the influx of marrow-derived MSCs capable of osteogenic differentiation [23]. Shirai et al. [24] reported an 88.2% healing rate using curettage with decompression through a cannulated hydroxyapatite pin, while Dormans et al. [25] achieved 91.7% healing with curettage, decompression, and calcium sulfate grafting. Hou et al. [26] reported a 91.6% success rate when curettage was paired with maintained medullary cyst continuity, outperforming curettage alone. The 15.0% reoperation rate in our curettage group at the mean 7.8-year follow-up aligns favorably with these benchmarks; the longer follow-up of the present study captures more late recurrences than most prior series.

Regarding hardware use, our data contrast with reports by Zhou et al. [27] and Mavčič et al. [17], who proposed that intramedullary nailing may facilitate healing through a decompression mechanism analogous to curettage. In our cohort, hardware use was neither protective nor harmful (Fig. 4) and was actually more common in the MOTAI group, where a higher proportion of patients required additional surgery. Creating a hardware channel through the cyst, without systematic curettage and cyst-wall disruption, appears insufficient to replicate the biological environment achieved by open curettage. The distinction may lie in the thoroughness of cyst-lining removal and the volume of medullary canal exposed. These mechanisms, though biologically plausible, remain theoretical and warrant prospective validation.

The biological rationale for BMAC/DBM rests on the osteogenic potential of marrow-derived MSCs [[28], [29], [30]]. The osteoconductive and osteoinductive properties of DBM, first characterized by Urist and Strates [31], provide a scaffold that amplifies MSC-mediated bone repair. D'Amato et al. [28] found that BMAC combined with equine DBM produced significantly better healing than methylprednisolone injection (83.3% vs 58.6% *P* = .047) in 53 children. Early series by Lokiec et al. [19] demonstrated encouraging outcomes with percutaneous BM grafting, and subsequent work showed that enriching BMAC with DBM further enhances cyst healing [20,21,32,33]. Additional evidence from Zamzam et al. [34] and Wright et al. [35] reinforces the role of marrow-derived progenitors. Together, these data outline a strong biological justification for BMAC/DBM, which our study does not challenge, but they also confirm that the surgical context in which this therapy is delivered is equally important.

The larger-bore core trocar used for the MOTAI technique, rather than a standard BM aspiration needle, was selected primarily to allow adequate decompression and drainage of cyst fluid and to permit instillation of the combined BMAC/DBM mixture, since DBM is a particulate/paste material rather than a liquid; a marrow needle could deliver BMAC alone but would not readily accommodate DBM delivery. This equipment choice reflects the requirements of the delivery method rather than a preference for BMAC over unconcentrated BMA, and a direct comparison of BMAC vs BMA remains outside the scope of the present study.

Despite the higher reoperation rate in the MOTAI group, final radiographic and functional outcomes were equivalent at a mean 7.8-year follow-up, with no significant difference in radiographic outcome (70.0% vs 73.3% good Neer results *P* = 1.00) or composite functional score (8.65 vs 8.27 *P* = .836). MOTAI alone does not preclude eventual healing but achieves it at the cost of significantly more surgical procedures. For patients and families, this has important implications regarding the number of anesthetics, hospitalizations, and rehabilitation episodes and should be incorporated into preoperative counseling.

The regression models reinforce these conclusions. Model A confirmed curettage as the only independent predictor of avoiding reoperation (OR: 9.92), with cyst activity failing to reach significance (OR: 2.14 *P* = .394). This is notable because active cysts were over-represented in the MOTAI group (73.3% vs 25.0%); controlling for this imbalance only strengthened the curettage effect. Model B confirmed that surgical approach, age, hardware use, cyst activity, or anatomical site did not predict the final radiographic outcome, consistent with the observation that UBCs tend toward eventual healing regardless of the chosen approach but that curettage shortens that path considerably.

Although curettage was associated with a significantly lower reoperation rate after adjustment for the 2 identified baseline imbalances, this finding should be considered in the context of the nonrandomized design and the possibility of residual confounding by indication. A larger, ideally multicenter or prospective, cohort would help balance baseline lesion characteristics between groups and yield more robust and precise multivariable estimates.

### Limitations

Several limitations should be considered. The retrospective, nonrandomized design introduces potential selection bias; however, group allocation was based on clinic availability rather than lesion characteristics and the multivariable analysis adjusted for the 2 baseline imbalances identified. Specifically, group assignment was determined by which surgical team or clinic a patient was scheduled with at presentation, reflecting scheduling and treating team availability, rather than a documented referral pathway based on lesion severity, size, or activity. Granular, prospectively recorded data on referral patterns between the 2 teams are not available, and although the multivariable models adjusted for the 2 identified baseline imbalances (active cyst status and hardware use), this does not fully exclude residual confounding by indication related to unmeasured differences in lesion aggressiveness between groups. The modest cohort size (*n* = 35) limited statistical power for subgroup analyses. Although the primary outcome reached statistical significance, multivariable estimates had wide CIs and should be interpreted in the context of the limited sample size. Specifically, the primary-outcome effect estimate (OR: 9.92, 95% CI: 1.27-77.35) illustrates this imprecision, and we caution against overstating the precision of these estimates when interpreting the findings.

The Neer classification has recognized interobserver variability; we attempted to minimize this by applying uniform grading criteria. The precise extent of curettage and degree of canal opening can vary subtly between surgeons and were difficult to quantify retrospectively.

Prospective, randomized studies with standardized surgical protocols are needed to confirm the role of curettage and the specific contribution of different biological adjuncts.

## Conclusion

Open curettage combined with BMAC/DBM injection significantly reduces the need for additional surgical interventions compared with MOTAI, without compromising the final radiographic healing or functional outcome at long-term follow-up. Curettage may be the principal surgical determinant of treatment efficacy in this setting, independent of cyst activity or hardware use. Hardware placement alone, without curettage, did not reduce reoperation risk, suggesting that mechanical preparation of the cyst environment is not replicated by implant-based decompression. These findings should be interpreted in light of the nonrandomized design and baseline differences between groups and warrant confirmation in a larger, prospective cohort.

## Additional links

• POSNAcademy: https://www.posnacademy.org/media/Unicameral%20Bone%20Cyst/0_0dyb83i0
• POSNAcademy: https://www.posnacademy.org/media/Bone%20Cysts%3A%20Principles%20of%20Care/1_pu19xh8w

## Author contributions

**Roy Gigi:** Writing – review & editing, Writing – original draft, Conceptualization.

## Ethics approval and consent

This study was approved by the Institutional Review Board/Ethics Committee of Tel Aviv Sourasky Medical Center (Ichilov Hospital), Tel Aviv, Israel. The requirement for written informed consent was waived owing to the retrospective nature of the study.

The authors declare that no patient consent was necessary as no images or identifying information is included in the article.

## Declaration of the use of generative AI and AI-assisted technologies in the writing process

During the preparation of this work, the authors used a large language model ChatGPT (OpenAI OpCo, LLC, 1455 Third Street, San Francisco, California 94158, USA) to assist with language editing and to check the manuscript for adherence to *JPOSNA*’s submission requirements. After using this tool, the authors reviewed and edited the content as needed and take full responsibility for the content of the published article. No generative AI tool was used to generate scientific content, analyze data, or produce figures.

## Funding

This research received no specific grant from any funding agency in the public, commercial, or not-for-profit sectors. No funding was received from the National Institutes of Health, the Wellcome Trust, the Howard Hughes Medical Institute, or any other organization in connection with this work.

## CRediT authorship contribution statement

**Roy Gigi:** Writing – review & editing, Writing – original draft, Conceptualization. **Amir Sternheim:** Conceptualization, Resources, Validation, Writing – review & editing. **Omri Merose:** Conceptualization, Writing – review & editing. **Adrian Tudor:** Conceptualization, Writing – review & editing, Validation. **Ortal Segal:** Conceptualization, Data curation, Supervision, Writing – review & editing. **Baruch Danino:** Conceptualization, Data curation, Methodology, Writing – review & editing. **Yair Gortzak:** Conceptualization, Methodology, Supervision, Writing – original draft, Formal analysis.

## **Declaration of competing interests**

The authors declare that they have no known competing financial interests or personal relationships that could have appeared to influence the work reported in this paper.
